# Globus pallidus internus activity increases during voluntary movement in children with dystonia

**DOI:** 10.1016/j.isci.2023.107066

**Published:** 2023-06-07

**Authors:** Estefania Hernandez-Martin, Maral Kasiri, Sumiko Abe, Jennifer MacLean, Joffre Olaya, Mark Liker, Jason Chu, Terence D. Sanger

**Affiliations:** 1 Department of Electrical Engineering and Computer Science, University of California, Irvine, Irvine, CA, USA; 2 Department of Biomedical Engineering, University of California, Irvine, Irvine, CA, USA; 3 Department of Neurosurgery and Neurology, Children’s Hospital of Orange County (CHOC), Orange, CA, USA; 4 Department of Neurosurgery, University of Southern California, Los Angeles, CA, USA

**Keywords:** Neurology, Pathophysiology, Neuroscience

## Abstract

The rate model of basal ganglia function predicts that muscle activity in dystonia is due to disinhibition of thalamus resulting from decreased inhibitory input from pallidum. We seek to test this hypothesis in children with dyskinetic cerebral palsy undergoing evaluation for deep brain stimulation (DBS) to analyze movement-related activity in different brain regions. The results revealed prominent beta-band frequency peaks in the globus pallidus interna (GPi), ventral oralis anterior/posterior (VoaVop) subnuclei of the thalamus, and subthalamic nucleus (STN) during movement but not at rest. Connectivity analysis indicated stronger coupling between STN-VoaVop and STN-GPi compared to GPi-STN. These findings contradict the hypothesis of decreased thalamic inhibition in dystonia, suggesting that abnormal patterns of inhibition and disinhibition, rather than reduced GPi activity, contribute to the disorder. Additionally, the study implies that correcting abnormalities in GPi function may explain the effectiveness of DBS targeting the STN and GPi in treating dystonia.

## Highlights

• A decreased thalamic inhibition in dystonia by abnormal patterns GPi activity
• Stronger coupling between STN-VoaVop and STN-GPi compared to GPi-STN
• Frequency pattern in dystonia with cerebral palsy at movement is in beta band

## Graphical abstract

**Figure:**
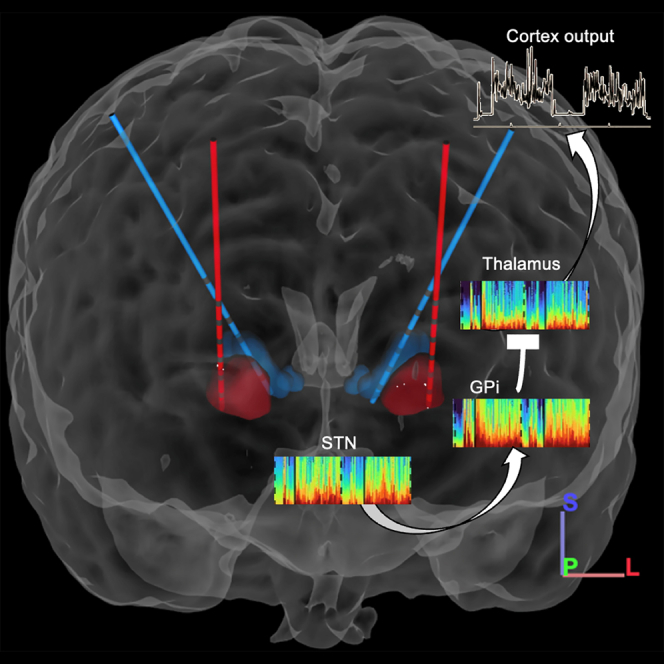

## Introduction

Dystonia in children is defined as a movement disorder in which involuntary sustained or intermittent muscle contractions cause twisting and repetitive movements, abnormal postures, or both.^1^ The mechanism underlying childhood dystonia is not fully understood but may include an imbalance between midbrain dopaminergic and striatal cholinergic signaling,^2^ abnormal patterns of subcortical activity, excessive basal ganglia or peripheral loop gain, or decreased focusing of intended patterns of muscle activity.^3^^,^^4^^,^^5^ Dystonia has been identified as the second most common movement disorder in pediatric patients with CP,^6^ impacting motor function, and causing pain and discomfort. Cerebral palsy (CP) encompasses a heterogeneous group of developmental disorders with a movement disorder pattern that is diagnosed as dyskinetic CP.^7^ Clinical goals for the treatment of dystonia involve maximizing function,^8^ with some non-invasive treatment options including enteral medications such as anticholinergics or muscle relaxants.^9^

Moreover, an overall improvement in patient outcomes has been achieved with deep brain stimulation (DBS), a method that uses implanted electrodes to modulate brain activity and signal transmission. In addition to stimulating, some leads (e.g., stereoelectroencephalography [sEEG] leads) offer the opportunity to record from human deep brain regions, where it can detect neural activity such as the brain wave frequencies that can be used as a physiomarker.^10^ A large number of studies have reported low-frequency oscillations (LFO) in theta and alpha bands in the globus pallidus internus (GPi) and the subthalamic nucleus (STN) for dystonia and Parkinson’s disease; moreover, increased beta band activity has been noted in both diseases.^11^ In contrast with previous studies that have focused solely on basal ganglia (GPi or STN), we recorded from thalamic subnuclei and basal ganglia simultaneously in awake pediatric patients with dystonia, as it has been recent informed to ameliorate the symptoms in adults.^12^ While the pallidum (GPi) has become the main DBS target for the treatment of dystonia in children,^13^^,^^14^^,^^15^^,^^16^ the response rates in dyskinetic CP are modest and unpredictable, with almost half of children responding poorly.^17^ Due to the limitations of pallidal DBS in dystonia, there is a need to explore alternative brain targets such as thalamic nuclei^18^ or the STN,^19^ which are rarely targeted and thus underexplored. Selection of targets may be aided by electrophysiological data recorded from temporary (sEEG) electrodes placed at target regions. We hence collected recordings from temporary deep brain electrodes in a new clinical procedure^20^^,^^21^ which provided us the opportunity to examine LFOs across multiple deep brain regions in awake patients.

Multiple deep brain recordings allow us to not only measure the activity patterns associated with dystonia, but also understand the physiology underlying the disorder. For example, the classical basal ganglia model explains how the flow of information is regulated by deep brain regions, and it is separated into “direct” and “indirect” pathways.^22^^,^^23^^,^^24^^,^^25^ In this model, the cortico-striato-GPi path is the “direct” pathway that facilitates movements, whereas the cortico-striatal-globus pallidus externus (GPe)-STN-GPi “indirect” pathway suppress movements. This classical model has been accepted as a model for normal function and several movement disorders including parkinsonism and dystonia.^25^^,^^26^ A prediction of the model has been confirmed in non-human primates is that there is normally a high-tonic firing rate of GPi-output neurons at rest in order to suppress thalamic neurons and inhibit signals to the motor cortex that would drive movement. The model predicts that GPi output should be paused or reduced in order to perform movement, allowing activation of thalamus.^23^^,^^27^^,^^28^

For dystonia, a commonly applied model is the “rate model” of basal ganglia, which predicts that a loss or lowering of excitatory output from the STN to GPi would lower inhibitory output from GPi to the thalamus and result in excessive involuntary movements.^29^^,^^30^ Several experimental and clinical results have been at odds with this prediction, including the improvement of dystonia with pallidotomy. Furthermore, a recent study noticed increased GPi activity during movement in two dystonic patients.^31^ These and other results have led several authors to suggest that the pattern of output from GPi in dystonia is more important than the overall average firing rate, so that an inappropriate or insufficient pattern of inhibition is a cause of dystonia.^24^^,^^32^^,^^33^^,^^34^ It is not known if these considerations apply to children with dyskinetic CP.

In order to understand the GPi function in dyskinetic CP patients, the ventral oralis anterior/posterior, VoaVop of the thalamus, GPi, and STN were recorded simultaneously in a group of awake children while either at rest or executing active voluntary movements with their upper limbs.

## Results

### Frequency patterns

**Figure 1:**
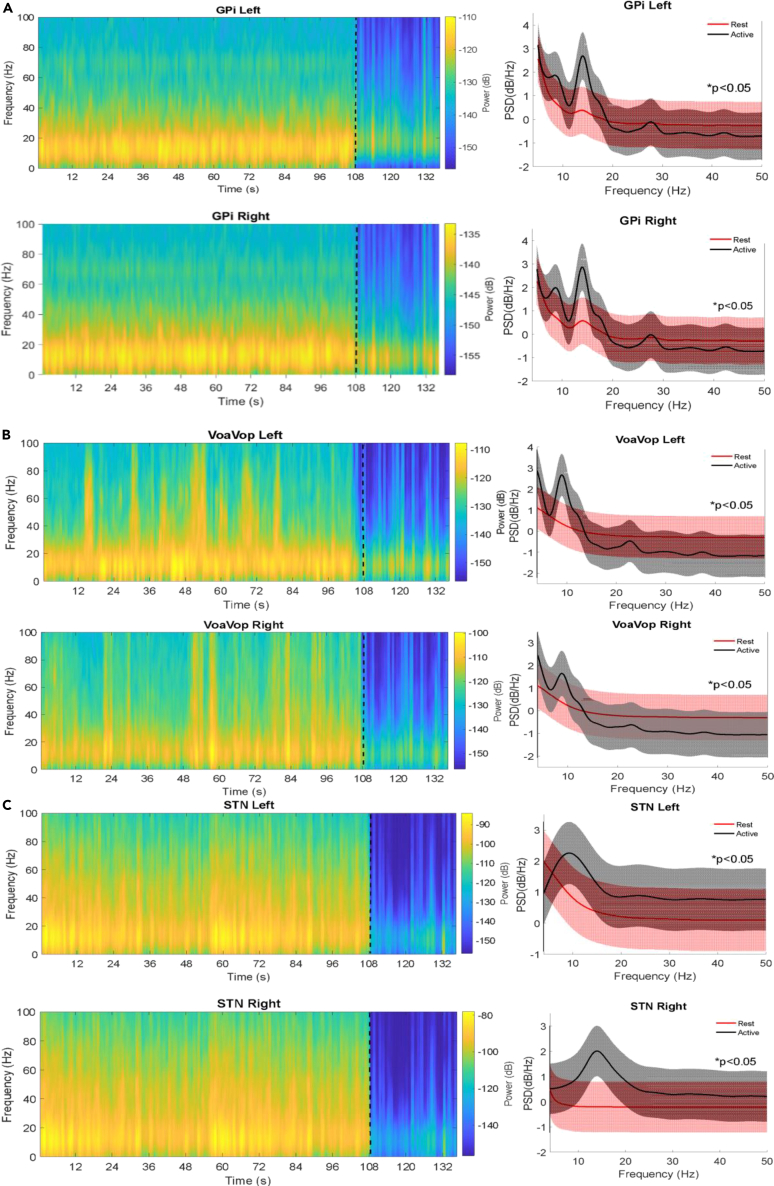
Power spectra analysis across the targets (A) Power spectra analysis of pallidum recordings. Spectrograms (left side) depict the power (dB) for the grand average averaged for movement-108s and resting-30s conditions, across all patients. Dotted black line separates movement (left) from resting (right). Vertical axis shows the frequency (Hz) and the horizontal axis is time (s). Power spectral densities (PSDs, right side) show the grand average across all patients. Red lines and black lines represent the resting and movement conditions, respectively; with a significant difference in spectra, ∗p value<0.05. PSDs show peaks in the theta (4–12 Hz) and beta (13–35 Hz) bands in the pallidum during contralateral voluntary movements executed with (top) right upper limb and (bottom) left upper limb. The vertical axis is power (dB/Hz), and the horizontal axis is frequency (Hz). (B) Power spectra analysis of thalamic recordings. (C) Power spectra analysis of subthalamic nuclei (STN) recordings.

Figure 1A shows the GPi spectrogram during rest and during movement of the contralateral upper limb. Spectrograms represent the pallidum recordings for both conditions (epoch-108s and epoch-30s), analyzed in time-frequency domain. During contralateral movements, the major power is clearly represented in the beta-band (13–35 Hz) for both cerebral hemispheres. PSD peaks were observed at low frequencies in the theta-band (4–12 Hz) on both cerebral sides; however, clear peaks were observed in the low beta-band with a maximum peak at 13.92 ± 0.11 Hz during voluntary movements with the contralateral arm. In contrast, recordings obtained during the resting condition show only smoothed peaks around 13 Hz for both cerebral sides, with much less power than that observed during movement. Both PSDs as well as the spectrograms show significant differences within theta-beta-band (p < 0.05, FWE corrected) between conditions (resting vs. movement) for pallidum recordings.

Video S1. A patient with cerebral palsy performing a voluntary reaching movement, related to STAR Methods

As performed for pallidum recordings, the thalamic recordings were obtained for both conditions (epoch-108s and epoch-30s, corresponding to movement and rest, respectively) (Figure 1B). Spectrograms show that major power is also in the beta-band (13–16 Hz). In addition, thalamic PSDs show smoothed peaks in the beta-band (13.99 ± 0.18 Hz) during contralateral movements, although these were smaller than those observed in pallidum (relative difference ∼1.34 dB/Hz). Also, thalamic recordings did not contain the theta-band peaks observed in pallidum. Another difference between the thalamic and pallidum recordings is the absence of peaks at any frequency band in thalamic recordings during resting conditions. Therefore, there are significant differences (p < 0.05, FWE corrected) between resting and the voluntary movement conditions in VoaVop.

Figure 1C depicts the subthalamic nucleus recording for both conditions. PSDs show peaks in a wide bandwidth of frequency, although the peaks in beta-band are dominant. In power terms, peaks in subthalamic nuclei were greater than those of thalamus, in both conditions and both cerebral sides (relative difference ∼0.7 dB/Hz). These peaks are also shown in spectrograms; as observed for thalamic recordings, a greater bandwidth of frequencies >30 Hz is observed, mostly on the right cerebral side. The resting conditions do not show peaks associated with any frequency band for any cerebral side, providing significant differences (p < 0.05, FWE corrected) between the two conditions. Moreover, contralateral recordings’ peak magnitude was higher in both pallidum and subthalamic nuclei recordings, with thalamic recordings.

**Figure 2:**
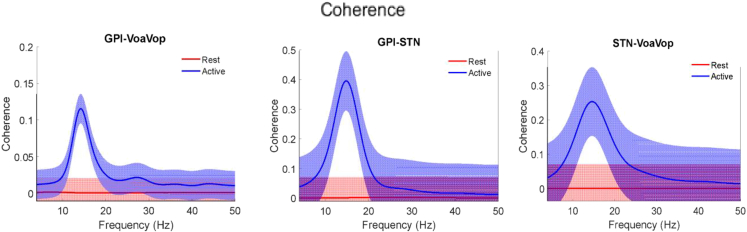
Coherence analysis Depicts the relationships between signals as a coherence function on the spectra for GPi-VoaVop, GPi-STN and STN-VoaVop.

The relationships between signals are represented by a coherence analysis (range of values from 0 to 1). Figure 2 shows the coherence for both cerebral sides (average ±SD) within 0–50 Hz; from GPi to VoaVop with peak coherences at ∼0.15 and notable differences from resting signals. In contrast, STN-VoaVop and STN-GPi show peaks coherences with values of approximately 0.3 and 0.5, respectively.

### Functional connectivity and its modulation by movement interference

**Figure 3:**
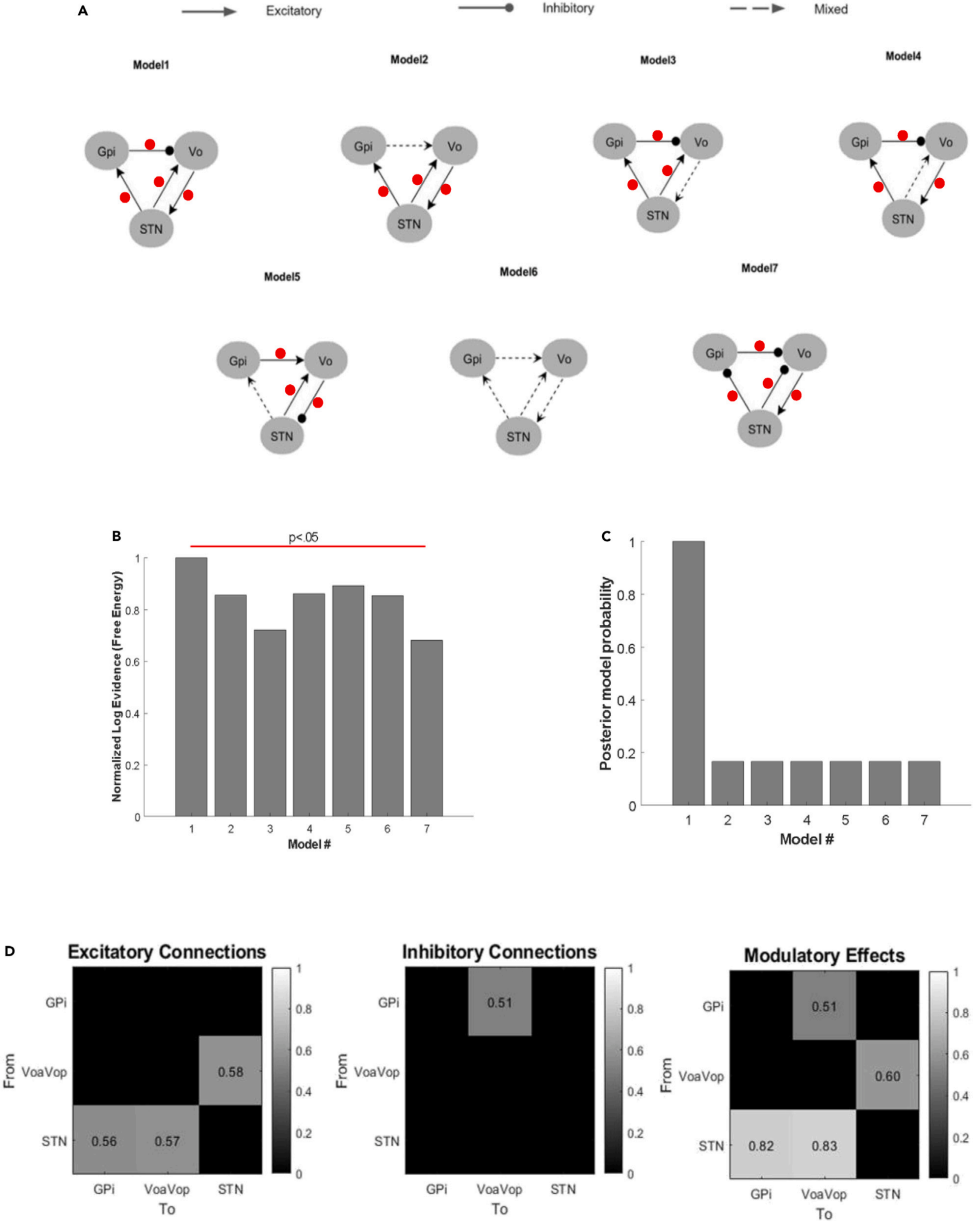
Network architecture of the basal ganglia-thalamus subnuclei loop (A) Schematic representations of the 7 models compared in this study. Models are illustrated in sets, with functional connectivity (arrows) expressed as excitatory, inhibitory, or mixed (excitatory and inhibitory) projections. Red dots indicate movement effects on each connection (whether the connection strength is modulated by movement vs. rest). (B) All models were compared using the free energy estimate of model evidence (p < 0.05). (C) Posterior model probability over models to corroborate the best model. (D) The strongest functional excitations are from the VoaVop to STN, from STN to GPi, and from STN to VoaVop. In terms of functional inhibition, the most prominent is from the GPi to VoaVop. Movement effect on connections (red dots), had the largest effects from STN to VoaVop, and to GPi (p > 0.8).

In order to study the relationship between the nuclei, DCM was computed. Although the cortex-basal ganglia-thalamic anatomic connectivity is widely known, we sought to test the transmission of information through this loop to determine which connections are more affected by the voluntary movement. To do this, we tested a total of seven possible connectivity models with inhibitory, excitatory, and mixed excitatory and inhibitory projections (Figure 3A). Models were selected to encompass several plausible connections between structures. Note that connections represent flow of coherent information but not necessarily a direct or monosynaptic connection between regions. For example, there may not be a direct anatomic connection between STN and VoaVop, although functional connectivity could be mediated through regions connected to both nuclei such as pallidum, or cortex. We included movement effects (how movement affects the connectivity gains). In order to estimate the functional connectivity using DCM, the model’s connectivity structure and parameter values constitute the system’s transfer functions, which are used to generate predicted cross-spectra between regions, for comparison with the observed spectra.

Based on the assumption that free energy estimates yielded valid results^35^^,^^36^ the first model was the best model based on the highest log-evidence (Figure 3B). The other models also showed high log-evidence, which reflects the goodness of the fit (p < 0.05). However, the first model described the data significantly better than other models (p < 0.05). We computed the *a posteriori* probability of each of the models to confirm the best-fit model.^37^^,^^38^ Based on this, the best model among those seven models was selected. It can be seen immediately that the first model has been selected (Figure 3C). Besides, the conditional probabilities of the interactions of movement on the functional connectivity were computed (0–1 values, gray scale). The strongest functional excitations (connections) are from VoaVop to STN, from STN to GPi, and from STN to VoaVop, while the most prominent functional inhibitions (connections) are from GPi to VoaVop as is expected according to the classical rate model. In terms of the movement interactions on these functional connections, the major effects of movement on these connections (probability >0.8) are on the STN-VoaVop and STN-GPi (Figure 3D).

**Figure 4:**
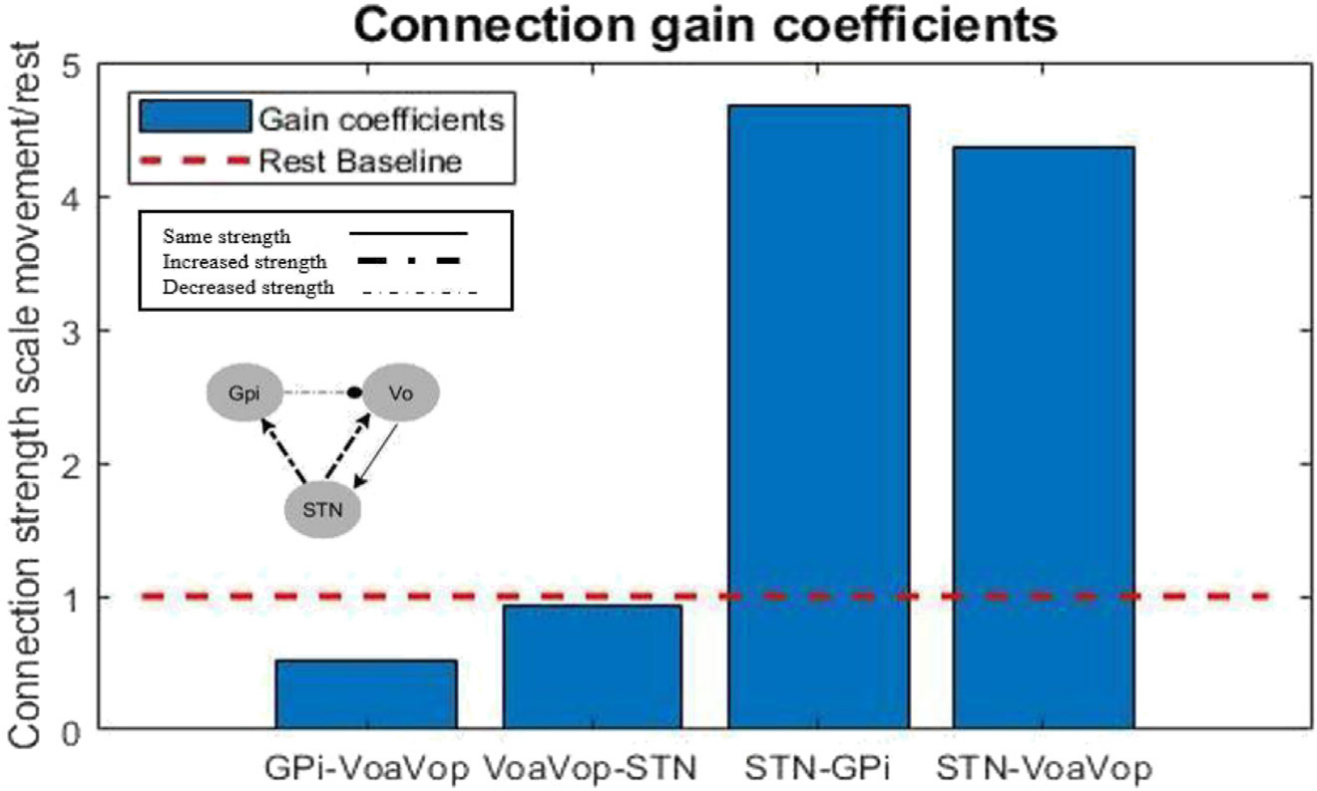
Estimation of coupling strength among the three structures analyzed for the first model presented, in terms of gain coefficients Gain coefficients were normalized to the resting state (red dotted line) to predict the movement effect with respect to the resting state. The effect of movement on functional connectivity is represented, with major effects observed as increased strength (black dotted lines) from STN to VoaVop and from STN to GPi. Movement was observed to decrease the functional inhibition strength between GPi and VoaVop.

The movement effect on the functional connections can also be interpreted as a change (increase or decrease) in the amount of energy transmitted between two locations which is quantified by the transfer functions’ gains. This value explains how the energy flow between two brain regions changes. Using the first model, we calculated the gain coefficients (here range of values from 0 to 5) to determine the connection strength changes during voluntary movement. The gain obtained from the movement state was normalized to resting state gains (defined with the value = 1), showing increase or decrease from the resting state for each functional connection (Figure 4). The strongest effects occur from STN to VoaVop and from STN to GPi, with values up to 4 times higher than those for resting state. Weaker effects are shown from GPi to VoaVop or from VoaVop to STN, with values below the resting state.

## Discussion

The basal ganglia compose a complex system that sends and receives feedback to the cortex through the thalamus and striatum. The STN and the striatum receive cortical inputs, while the GPi sends information to the thalamus, which modulates cortical activity. The “rate model” of basal ganglia^24^ suggests that high activity in GPi is required in order to inhibit movement at rest. In particular, the rate model postulates that increased movement in dystonia is due to low-firing rates in GPi. However, alterations of this circuit resulting in dystonic muscle contractions are still not understood. Our surgical procedure^20^ allows us to simultaneously record from multiple targets in awake patients without the influence of anesthesia. Therefore, it allows us to record valuable and useful data and answer some questions such as: 1) which frequency bands are associated with movement for each targeted deep brain region in dyskinetic CP. 2) are the results consistent with predictions of the “rate model” of basal ganglia in dyskinetic CP.

### Frequency patterns in dyskinetic CP

A characterization of frequency patterns in dyskinetic CP is fundamental to understand the underlying dysfunction in children with dystonia. Although many previous studies have focused on frequency patterns in pallidum in patients with clinically successful outcomes,^39^^,^^40^^,^^41^^,^^42^ other results that have elucidated the pathophysiology underlying dystonia include bursting cells in the GPi (discovered from single-unit recordings during DBS surgery^43^ and theta band activity in GPi^10^ and the STN in patients with dystonia.^44^ Nonetheless, the action mechanism of pallidum in the pathophysiology of dystonia is currently under debate. One hypothesis suggests that dystonic muscle contractions can be caused by excessive gain in the cortex-basal ganglia-thalamic loop, with related effects on motor network function.^45^ There is also growing evidence for the involvement of other brain regions, including the cerebellum^31^^,^^46^ and sensorimotor cortex.^47^ The presence of peaks in both beta and theta bands in pallidum is likely to be significant to the pathophysiology of dystonia. In alignment with previously reported findings,^10^ our results show activity in the theta band; however, across patients, the peak identified most clearly during voluntary movement was contained within the low beta band (maximum peak ∼13 Hz). Since STN receives input from the cortex via the hyperdirect pathway, we expected to find power in a wide frequency range during movement, and this hypothesis was confirmed by our results (Figure 1C). Beta-band peaks were also observed in thalamic subnuclei, albeit with magnitude less than that of peaks observed in pallidum, with detectable differences from the peaks observed during resting. Although the major power is in the beta band, thalamic recordings also show power at higher frequencies (>30 Hz) (Figure 1B). As observed in pallidum and thalamus, the major power in STN is concentrated in the beta band. These results lead us to think that the signal transmission generated in the beta band (∼13 Hz) by movement is maintained throughout all structures from STN to GPi and from GPi to VoaVop, with detectable differences in magnitude between areas of connectivity, as is shown in Figure 2.

Based on the basal ganglia model, the GPi-VoaVop connection should have the highest magnitude with negative (inhibitory) correlation,^48^ however, Figure 2 shows that the strongest connections are between GPi-STN and STN-VoaVop. On the one hand, the basal ganglia GPi-STN contain higher magnitudes than thalamus during movement; therefore, the basal ganglia are modulating the thalamic output.^49^ On the other hand, although there is no direct anatomic connection between STN and VoaVop, their coherence has higher magnitude than GPi-VoaVop. Therefore, the results are inconsistent with the theory of thalamo-cortical disinhibition due to decreased output from GPi.^24^

### Functional connections and their modulation by movement interferences

Our results (Figure 3) confirm current hypotheses of classical model of basal ganglia. In particular, we have confirmed the inhibitory connection of pallidum on thalamus, and we have also confirmed the expected excitatory effect of STN on pallidum. More surprisingly, we have shown a strong bidirectional functional excitation between STN and motor thalamus. This functional excitation is clearly shown by the results from the free energy estimates (Figures 3B and C). Additionally, the results are consistent in explaining how voluntary movement is associated with changes in the transmission of information through the basal ganglia and thalamus (Figure 4). This functional excitation is probably mediated through other brain regions including cortex since there is currently no evidence for a direct excitatory connection from STN to thalamus in humans.

**Figure:**
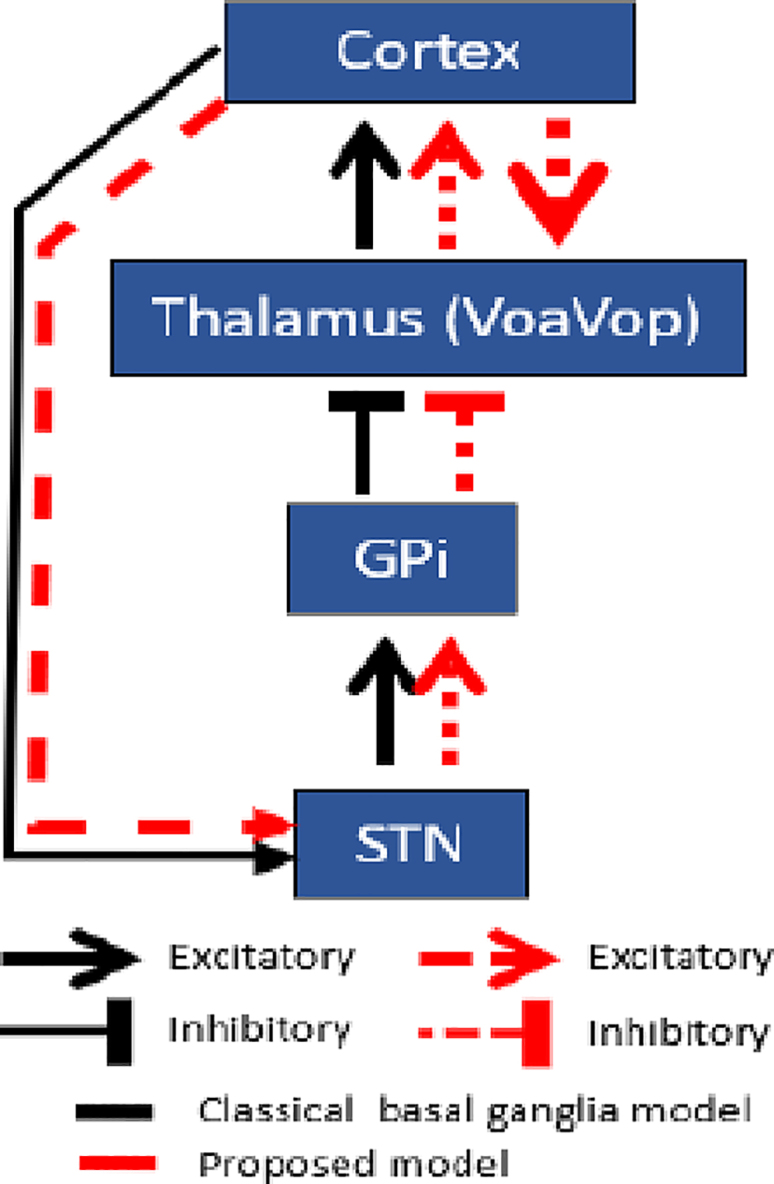

Assuming that there is not a direct anatomic connection between STN and VoaVop, we conjecture that there is inappropriate thalamic excitation as a result of an input from the cortex, which in turn activates downstream STN via the hyperdirect pathway, increasing STN activity. STN in turn excites GPi, resulting in increased activity in GPi during movement but not high enough to inhibit the existing thalamus excitation. GPi outputs “sculpt” ongoing activity in thalamus, providing a functional increase in both thalamus and STN (red loop) through cortex inputs.

Recordings from nonhuman primates and Parkinson’s disease have been consistent with the hypothesis of high-GPi activity at rest.^23^^,^^27^^,^^28^ Our results differ significantly, as previously reported.^31^ One possible explanation is that the function and connectivity of GPi are significantly different in children with dyskinetic CP. Another possibility is that the nature of our recordings, performed in pediatric patients that are unrestrained and comfortable in a hospital bed, allows for true relaxation, whereas nonhuman primates are not in fact at rest while restrained in an electrophysiology recording apparatus. It is also possible that the high levels of activity seen in Parkinson’s disease^49^ represent a significant abnormality that is not present in dystonia.

We observed strong movement-related modulations both STN and VoaVop, despite the lack of evidence for direct interactions between these two nuclei from animal models.^50^ Excitatory inputs from the motor cortex may explain these results and suggest a mechanism for effectiveness of deep brain stimulation in STN^51^ and GPi^41^ to treat dystonia.

The DCM analysis confirms the inhibitory connection between GPi and motor thalamus. In addition, our results from frequency analysis clearly show increased activity in both regions during movement compared to during rest. We reconcile these two apparently contradictory findings by proposing that GPi inhibits a subpopulation of thalamic neurons, whereas most of the thalamic target neurons must be excited from other sources (probably cortical inputs). Therefore, our findings are consistent with the hypothesis that GPi outputs “sculpt” ongoing activity in thalamus and thereby perform precision modulation of the signal returning to motor cortical areas. This hypothesis is consistent with prior models of the control of motor patterns.^24^^,^^32^ Our results provide support for a more complex model of basal ganglia function that may be helpful to understand the mechanism of dystonia and offer potential new treatments.

### Limitations of the study

Our data do not allow us to compare the functional connectivity in the dystonic brain with that of a healthy brain, and thus we do not know which observed patterns are responsible for dystonic symptoms. Nevertheless, these patterns of activity and connectivity do indicate the frequency content and information flow within the dystonic brain, and the data are directly related to the motor function (both dystonic and non-dystonic) of our patients.

The results presented above do not allow for definite conclusions regarding the pathophysiology of childhood dystonia, or whether there might be other mechanisms that can cause dystonia. The discrepancies with other reports could be due to factors such as diverse forms of dystonia (postural deformities, OFF-dystonia, cervical, torticollis, etc.). However, the presence of exaggerated beta oscillations in the dystonic STN is in fact physically possible. The role of other factors such as different types of treatments might have contributed to discrepancy of results across studies (11).

Our results are limited by recordings from potential DBS targets. A full understanding of the role of basal ganglia and thalamus in motor control may require recordings from striatum, cortex, and brainstem targets, which may not be feasible in humans, but it will help the data interpretations. This fact has not been contemplated in the present study due to the difficulty to acquire healthy control data using the DBS technique.

Finally, our results provide information on the pattern of activity and functional connectivity between regions in basal ganglia and thalamus in children with dyskinetic CP. Further studies will be needed to determine which components of the observed activity are responsible for dystonia, which represent compensation for dystonia, and which represent normal patterns. Nevertheless, the importance of these regions as targets for deep brain stimulation implies that a detailed understanding of the patterns of deep brain activity can be helpful to elucidate the mechanism of DBS and improve the process of the clinical selection of appropriate targets.

## STAR★Methods

### Key resources table

**Table:** 

REAGENT or RESOURCE	SOURCE	IDENTIFIER
**Software and algorithms**		

MATLAB 2021a	MathWorks, USA	https://es.mathworks.com/products/matlab.html
SPM12	The Wellcome Trust Centre for Neuroimaging	https://www.fil.ion.ucl.ac.uk/spm/software/spm12

### Resource availability

#### Lead contact

• Further information and requests for resources and reagents should be directed to and will be fulfilled by the lead contact, Estefania Hernandez-Martin (estefania@sangerlab.net).

#### Materials availability

• All data are available in the manuscript text and supplemental information.

### Experimental model and study participant details

Nine pediatric patients undergoing deep brain stimulation (DBS) implantation for the treatment of dyskinetic CP were recruited to participate in the present study (Table S1). Due to the specific focus of our study on physiological recordings, gender or sex does not impact on the obtained results. All patients included in the study were of European ancestry. In each case, the diagnosis of dystonia was established by a pediatric movement disorder specialist (T.D.S.) using standard criteria.^52^ All patients provided signed informed consent for surgical procedures in accordance with standard hospital practice. The patients, or parents of minor patients, also signed informed consent for the research use of electrophysiological data and Health Insurance Portability and Accountability Act (HIPAA) authorization for the research use of protected health information. The patients are transferred to the neuromodulation unit (NMU) at the hospital for DBS programming and testing different patterns of stimulation and the assessment of their efficacy. At this stage, which takes place during 5 to 7 days, the children are awake, and we record the brain signals through depth electrodes. After two weeks from the temporary lead removal, the permanent leads are placed in the selected DBS targets as well as the implantable pulse generators.

### Method details

#### Surgical procedure

Our clinical procedure for determining DBS targets includes the implantation of 10 temporary AdTech MM16C depth electrodes (Adtech Medical Instrument Corp., Oak Creek, WI, USA) at potential DBS targets (including basal ganglia and thalamic subnuclei), as identified based on clinical criteria in each patient.^20^ Depth electrodes were placed using standard stereotactic procedures, with the most distal stimulation contact placed at the target location. Electrode location was confirmed by co-registration of the preoperative magnetic resonance imaging (MRI) with postoperative CT scan. Thalamic targeting was confirmed by identification of leads in subnuclei known to have greater or lesser response to median nerve electrical stimulation.^53^ For this study, we analyze data from electrodes in GPi, VoaVop, and STN. VoaVop is a known motor nucleus, while Vim is a sensory nucleus, and here we are focusing mainly on the motor pathways within the deep brain. Besides, VA (motor thalamic subnuclei) was not targeted for all patients, therefore, Vim and VA data was not analyzed as part of this study. Note the DBS implantation itself generates micro-lesions in all targets. No structural damage in the targets was reported in MRI of our cohort. Moreover, there were no significant perioperative adverse events.

#### DBS electrode localization

Preoperative T1-weighted (anatomy) volumes were acquired from a MAGNETOM 3 T (SIEMENS Medical System, Erlangen, Germany) MRI scanner for precise anatomical localization. Postoperative computerized tomography (CT) volumes were acquired for all patients from a GE (GENERAL ELECTRIC Healthcare, Milwaukee, WI, USA) CT scanner. Both the T1-weighted MRI image and the CT scan were co-registered and warped into MNI (Montreal Neurological Institute) space using ANT Advanced Normalization Tools.^54^ DBS electrodes were pre-localized in native and MNI space using the PaCER algorithm.^55^ A total of 9 patients each with bilateral VoaVop-STN and GPi DBS electrodes were visualized through DSI Studio using the DISTAL atlas^56^ (Figure S1. 3D rendering of group-based DBS electrodes for all patients). Note the VoaVop and STN recordings are from different contacts on the same MM16C electrode since the electrode trajectory permits entry of the STN with the distal contact and simultaneous entry of the VoaVop nucleus of the thalamus with proximal contacts.

#### Electrophysiological recording

Recordings were performed during the first 24 to 48 hours after clinical implantation of the temporary depth electrodes. Each electrode lead has a diameter of 1.2 mm and contains 6 low-impedance (1–2 kΩ) ring macrocontacts with 2 mm height and 5 mm on-center spacing, as well as 10 high-impedance (70–90 kΩ) microcontacts (50-μm diameter). The microcontacts are arranged in groups of 2 or 3, spaced evenly around the circumference of the electrode shaft, halfway between neighboring pairs of ring macrocontacts. The external proximal ends of the electrodes were connected to Adtech Cabrio™ connectors modified to include a custom unity-gain preamplifier for each microwire contact to reduce noise and motion artifacts. Macrocontacts bypass the preamplifiers to allow for external electrical stimulation. All data reported here are from the high impedance microcontact recordings, following our testing protocols that include stimulations using the macrocontacts, while the neural responses are recorded by the microcontacts. Microcontact electrode signals were amplified, sampled, and digitized by a Tucker-Davis Technologies PZ5M analog-to-digital amplifier connected to an RZ2 digital signal processor. Data was streamed to an RS4 high-speed data storage unit, controlled by Synapse recording software (System3, Tucker-Davis Technologies Inc., Alachua, FL, USA). The microcontact electrode signals were recorded from all implanted electrodes, sampled at 24 kHz, and stored for offline analysis.

All data were recorded while patients were awake sitting comfortably in bed. The voluntary reaching task protocol was explained to the patients, and if necessary, they were advised to practice prior to the main trials. They were then asked to perform the task with either of their upper limb while contralateral depth electrode signals were recorded.^57^ In all cases, dystonia was present in the tested limb, as evidenced by the presence of dystonic postures interfering with expected performance of the task. The reaching task consisted of 108 s of voluntary reaching followed by 30 seconds of resting period, for a total of four consecutive trials (Figure S2, Video S1). They repeated the reaching task with the other side as well. Some subjects could not complete the four trials due to fatigue and extreme dystonia during the movement, however all completed at least two trials of the reaching task experiment with each limb separately.

### Quantification and statistical analysis

#### Data analysis

All recordings (n=9; mean age= 13.6 ± SD=2.8) were processed in the MATLAB, MathWorks Inc. (2021a), Natick, Massachusetts. The unavoidable realities of mechanical, physical, and thermal noise decrease the signal-to-noise ratio (SNR), making it difficult to detect neural activity. Therefore, we used orthogonal multilevel wavelet decomposition (MWD)^58^ for signal filtering (MATLAB function “MODWT”). Before analysis, data portions that held obvious electrical artifacts were rejected after visual inspection. Information from bilateral GPi, VoaVop, and STN recordings during voluntary movement with the upper limbs (right and left) was obtained for all patients (n=9). Data recorded from those microcontacts placed within deep brain targets were used for the analysis in the frequency domain using fast Fourier transform based methods, (MATLAB function “fft”). The power spectra were normalized with respect to baseline (rest period) across all patients. Multitaper time-frequency spectrum continuous processes (MATLAB function “pmtm”) were also calculated. Resting periods that involved involuntary movements were rejected, after comparison with the surface electromyography (EMGs) recordings and voluntary movement portions, for each single patient (Figure S2). EMG signals of the task relevant muscles were also recorded and used to confirm the voluntary reaching task periods in the intracranial recordings for each patient individually. EMG recordings are not used for modeling in this study.

Grand averages were calculated for 108 seconds of voluntary reaching task and 30 seconds of resting periods across all microcontacts within brain targets represented by power spectrum densities (PSD). To study the coupling between deep brain nuclei and to rule out the notion that the peak power findings are not associated with movement artifacts, the coherence was calculated for both conditions. Coherence provides a frequency domain measure of the linear phase and amplitude relationships between signals and can reveal spectrally specific functional connectivity between neuronal populations.^59^ Data from both hemispheres and all targets (average ± SD) were used for the coherence analysis. The nonparametric Wilcoxon signed-rank test was used to compare the spectral features between the rest and movement conditions. The significance threshold was set at 0.05 with Bonferroni correction for multiple comparisons.

#### Dynamic causal modeling (DCM)

Dynamic causal modeling (DCM) implemented in Statistical Parametric Mapping (SPM12, The Wellcome Trust Centre for Neuroimaging, University College London) was used to model neuronal dynamics in each region as well as interactions within and between regions. In the study of local brain signals, DCM has elucidated the structure and synaptic properties within neuronal sources and the synaptic input that each source receives.^60^ Mean-field models (MFMs), a neuronal model variant of DCM for electrophysiological data, allow us to study the distribution of the neuronal population response based on a probabilistic representation of activity, which is encoded by the sufficient statistics of a probability density model. MFMs allow us to model the functional and effective connectivity between STN-VoaVop-GPi regions. This model allows us to understand how the connections are modulated during the transition from resting to movement states.^35^ The MFM was fitted to basal ganglia and thalamic data to model the neuronal activities and to perform the DCM. This allowed us to further estimate the transition parameters from resting to movement states.

## Data Availability

• The data reported in this study cannot be deposited in a public repository because are medical records. To request access, contact Terence Sanger (terry@sangerlab.net) or the lead contact. • This paper does not report original code. • Any additional information required to reanalyze the data reported in this paper is available from the lead contact upon request.
