# Supplementary material for: Globus pallidus internus activity increases during voluntary movement in children with dystonia
Source: iScience. 2023 Jun 7;26(7):107066. doi: 10.1016/j.isci.2023.107066 (PMC10300218; doi:10.1016/j.isci.2023.107066)
Supplement: Document S1. Figures S1 and S2 and Table S1 [file mmc1.pdf]

**iScience, Volume *26***

## Supplemental information

# Globus pallidus internus activity increases during voluntary movement in children with dystonia

**Estefania Hernandez-Martin, Maral Kasiri, Sumiko Abe, Jennifer MacLean, Joffre Olaya, Mark Liker, Jason Chu, and Terence D. Sanger**

**SUPPLEMENTAL INFORMATION**

| Patients | Etiology | Characteristics | Gender | Age | Leads |
|---|---|---|---|---|---|
| NMU1[a] | Dyskinetic CP (vasculitis) | Dystonia | M | 14 | GPi; Voa/Vop; STN; VIM; VPL |
| NMU2 | Dyskinetic CP | Dystonia | F | 12 | GPi; Voa/Vop; STN; VIM; VA |
| NMU3 | Dyskinetic CP (lymphangioma) | Dystonia | M | 20 | GPi; Voa/Vop; STN; VIM; VPL |
| NMU4 | Dyskinetic CP | Dystonia | F | 15 | GPi; Voa/Vop; STN; VIM; VA |
| NMU5 | Dyskinetic CP (XXX syndrome) | Dystonia | F | 10 | GPi; Voa/Vop; STN; VIM; VA |
| NMU6 | Dyskinetic CP (fetal distress at birth with cardiac arrest) | Dystonia | F | 12 | GPi; Voa/Vop; STN; VIM; VA |
| NMU7 | Dyskinetic CP (TUBB4A mutation) | Dystonia | M | 13 | GPi; Voa/Vop; STN; VIM |
| NMU8 | Kernicterus (G6PD deficiency) | Dystonia | M | 15 | GPi; Voa/Vop; STN; VIM; VA |
| NMU9 | Dyskinetic CP (variants of unknown significance in KMT2B and CACNA1A) | Dystonia | M | 12 | GPi; Voa/Vop; STN; VIM; VA |

**Table S1.** Demographic characteristics[a], related to STAR methods. Patients were identified from among the population of patients undergoing treatment in the neuromodulation monitoring unit (NMU) at Children's Health Orange County and Children's Hospital of Los Angeles. Abbreviations: CP, cerebral palsy; F, female; M, male; GPi, globus pallidus internus; Voa/Vop, ventral oralis anterior/posterior; STN, subthalamic nucleus; VA, ventral anterior; VPL, ventral posterolateral.

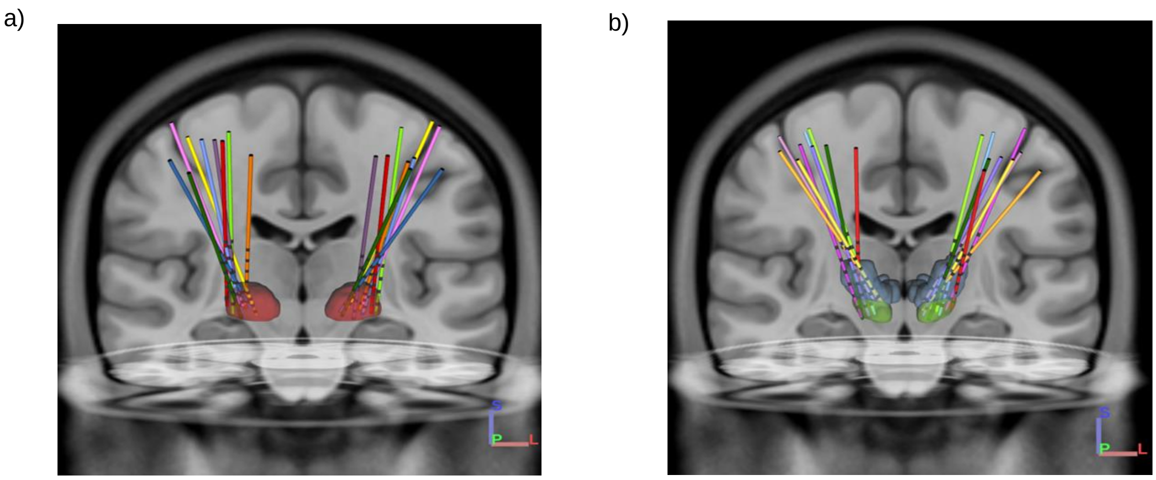


**Figure S1.** 3D rendering of group-based DBS electrodes for all patients, related to STAR methods. DBS electrodes in bilateral a) globus pallidum interna (GPi) and b) subthalamic nucleus (STN) covering the most distal region of the electrodes (green), with the remaining electrodes covering the ventral oralis anterior/posterior nuclei (blue). Coronal view from normalized scans into MNI space. Deep brain boundaries were defined with the DISTAL atlas. Each pair of DBS electrodes corresponds to a single patient, with each patient represented in a different color. A total of 18 bilateral DBS electrodes in 9 dystonic patients are represented through DSI Studio.

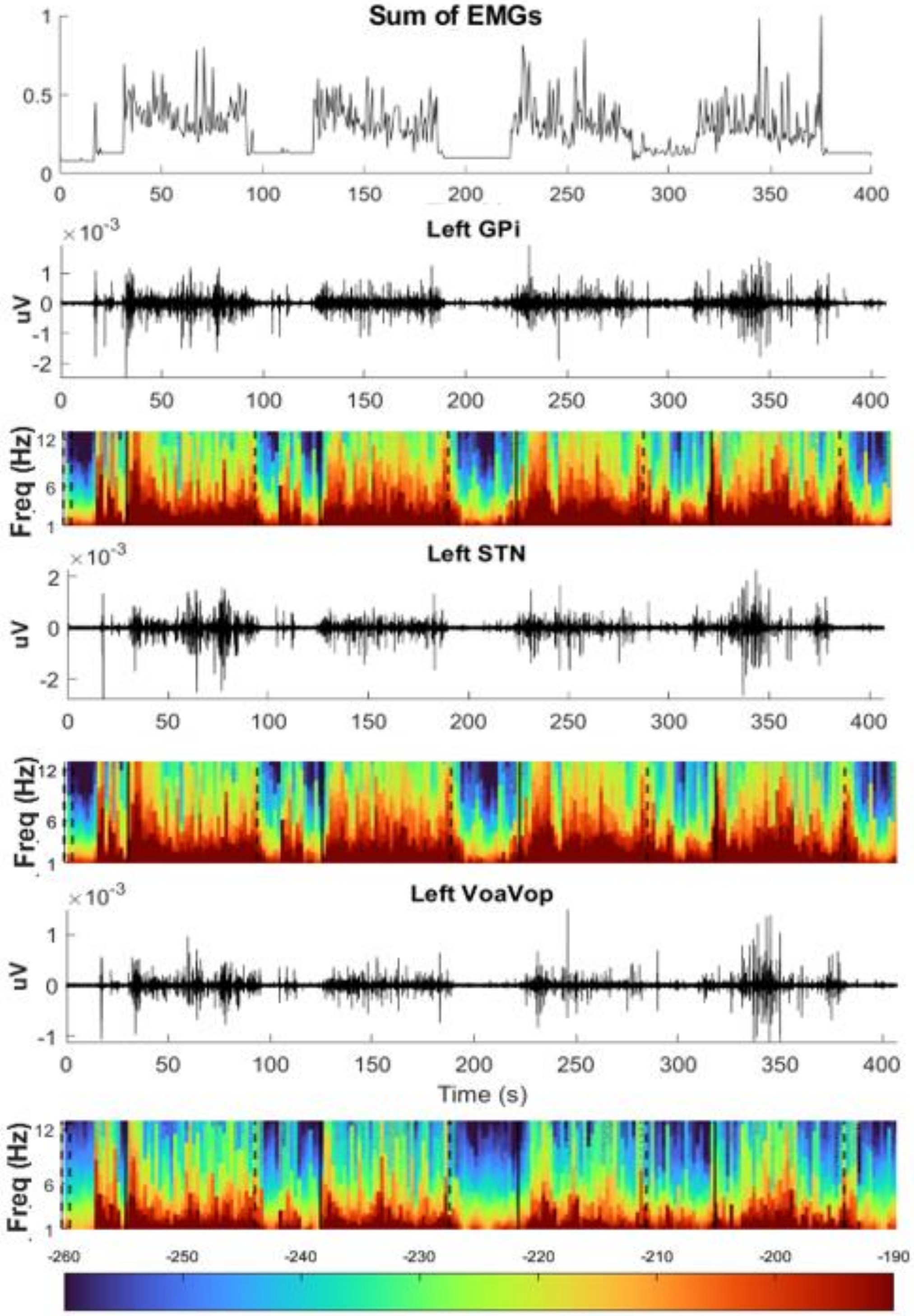


**Figure S2.** Individual EMG and intracranial recordings, related to STAR methods. Sum of the surface EMG recordings from the task relevant muscles (right biceps, triceps, wrist flexor, and wrist extensor) is shown at the top. An example of GPi, STN and VoaVop recordings during contralateral voluntary reaching task in one patient across four trials is also shown. Raw data (black lines) in each deep brain region illustrates the overall changes in activity. Spectrograms of the microwire electrodes placed in the GPi, STN and VoaVop show the increased power during voluntary movement (onset at vertical solid black lines) and at rest (onset at vertical dashed black lines). Horizontal axis shows time (s), and the vertical axis shows the frequency (Hz). Color bar shows the power range.
